# The Potential of NO_3_-N Utilization by a Woody Shrub Species Lindera triloba: A Cultivation Test to Estimate the Saturation Point of Soil NO_3_-N for Plants

**DOI:** 10.1100/tsw.2001.378

**Published:** 2001-11-20

**Authors:** Lina Koyama, Naoko Tokuchi, Muneto Hirobe, Keisuke Koba

**Affiliations:** Graduate School of Agriculture, Japan

**Keywords:** nitrate reductase activity (NRA), nitrate (NO_3_-N) concentration, perlite, Lindera triloba

## Abstract

Responses of seedlings of a shrub species, Lindera triloba, grown in perlite culture medium, to nitrate (NO_3_-N) supply were investigated to estimate the saturating point of available NO_3_-N for plant utilization. NO_3_-N concentration and nitrate reductase activity (NRA) in leaves and roots were used as indicators of NO_3_-N uptake and assimilation by L. triloba. Root NRA increased with NO_3_-N supply when concentrations were low and reached a plateau at high NO_3_-N concentrations. On the other hand, root NO_3_-N concentration increased linearly with NO_3_-N supply; therefore, it is suggested that NO_3_-N uptake did not limit NO_3_-N assimilation by L. triloba. In contrast, leaf NRA and leaf NO_3_-N concentration were low and were not influenced by NO_3_-N supply. This may be caused by the lack of transport of NO_3_-N from roots to leaves. The NO_3_-N retained in perlite was compared with NO_3_-N pool sizes in soils from a forest where L. triloba occurs naturally to estimate the level of NO_3_-N availability to plants in the forest soil. The maximum NO_3_-N pool size in the forest soil was comparable to concentrations at which root NRA reached a plateau in perlite cultures. These results indicate that soil NO_3_-N availability is below the saturation point for NO_3_-N uptake by L. triloba, and it is the limiting factor of NO_3_-N utilization by L. trilobaunder field conditions in which this species naturally occurs.

## INTRODUCTION

The increased nitrate ($NO_3^-$-N) deposition derived from human activities has altered ecosystem nitrogen (N) cycles and has increased N availability to plants. It could, therefore, reduce the diversity in ecosystems over the long term[1]. Under changing regional or global N cycles, $NO_3^-$-N uptake by plants is one of the most important processes in forest ecosystem N cycles. Because $NO_3^-$-N is a highly leachable anion in forest soils, plant $NO_3^-$-N uptake reduces not only N loss from ecosystem, but also other nutrient cations accompanied by $NO_3^-$-N leaching[2]; therefore, work is being conducted to elucidate the importance of plant $NO_3^-$-N use in N cycles in ecosystems[3,4,5], and information on the potential of plants for utilizing $NO_3^-$-N is needed to assess the roles of plants influencing N retention by ecosystems.

Regarding assimilation processes of $NO_3^-$-N by plants, the reduction of $NO_3^-$-N to $NH_4^+$-N is required for the synthesis of organic N[6,7,8]. The first step after the uptake of $NO_3^-$-N is the reduction of $NO_3^-$-N to nitrite ($NO_2^-$-N), and the process catalyzed by nitrate reductase (NR) is known to be the rate-limiting step in the sequence of $NO_3^-$-N assimilation processes[7,9,10]; therefore, plant nitrate reductase activity (NRA) is a useful indicator of plant $NO_3^-$-N utilization potential. Also, the existence of $NO_3^-$-N in plant tissues can be evidence for plant $NO_3^-$-N uptake, as plants do not synthesize $NO_3^-$-N[11].

The objectives of this study were to estimate the potential of $NO_3^-$-N use by plants and to determine whether the $NO_3^-$-N pool size in a forest soil exceeds plant $NO_3^-$-N uptake potential. For these objectives, we selected a shrub species of Lauraceae, *Lindera triloba* (Sieb. et Zucc.) Blume. *L. triloba* was one of the dominant understory species in a conifer plantation (Koyama, unpublished data), where nitrification potential had wide range (0 to 12.2 mg N 100 g dry soil$^{-1}$ 28 days$^{-1}$)[12]. Experiments were conducted (1) to describe the responses of $NO_3^-$-N use by *L. triloba* to $NO_3^-$-N supply and (2) to examine the relationship of $NO_3^-$-N supply to the amount of $NO_3^-$-N retained in the cultivation medium and to compare this with the $NO_3^-$-N pool size in forest soil. In seedlings of *L. triloba* grown in perlite medium supplied with various amounts of $NO_3^-$-N, leaf and root NRA were measured, in addition to leaf and root $NO_3^-$-N assays. The amount of $NO_3^-$-N retained in perlite was compared with the soil $NO_3^-$-N pool size in a forest where *L. triloba* is distributed.

## METHODS

### Plant Cultivation and Treatment

All seeds of *L. triloba* (Sieb. et Zucc.) Blume were collected from a single seed tree in Mt. Ryuoh in Shiga Prefecture, central Japan (35°10′N, 136°20′E) in September 1997. The collected seeds were stored at about 8°C until sowed in horticultural soil in April 1998. On April 29, 1999, seedlings were washed in tap water followed by deionized water to remove soil from roots. They were then individually transplanted into plastic pots filled with approximately 600 ml perlite that was prerinsed with deionized water. Throughout the period of the experiment, all seedlings were placed under a roof of a plastic film to keep out rain.

For 42 days after transplanting, each seedling was supplied daily with 200 ml nutrient solution containing 0.35 mmol l$^{-1}$ $NaH_2PO_4{\cdot}2H_2O$; 0.63 mmol l$^{-1}$ KCl; 0.5 mmol l$^{-1}$ $CaCl_2{\cdot}2H_2O$; 0.25 mmol l$^{-1}$ $MgSO_4{\cdot}7H_2O$; 59.37 µmol l$^{-1}$ Fe-EDTA; 0.43 µmol l$^{-1}$ Cu-EDTA; 0.42 µmol l$^{-1}$ Zn-EDTA; 0.45 µmol l$^{-1}$ Mn-EDTA; 32.35 µmol l$^{-1}$ $H_3BO_3$; 0.41 µmol l$^{-1}$ $Na_2MoO_4{\cdot}2H_2O$, and $NO_3^-$-N. Nitrate was added in solution as $NaNO_3$ at 0, 1, 10, 25, and 50 ppm (molar concentrations were 0, 0.071, 0.71, 1.79, and 3.57 mmol N l$^{-1}$). Each of the five treatments was replicated ten times.

### Plant Analysis

The leaves and roots of cultivated *L. triloba* were collected from 10:00 to 14:00 on June 8, 1999 at the 42nd day after the start of $NO_3^-$-N additions. NRA was measured by a modified version of the *in vivo* test[13,14,15,16]. Samples were kept at 4°C until laboratory analysis. Two hundred leaf disks each with a diameter of 2.5 mm were cut out, and fine roots (diameter < 2 mm) were cut into about 5-mm lengths after being rinsed with deionized water. After vacuum infiltration (6 mm Hg; twice for 30 s each) with 5 ml of incubation buffer, the samples were incubated for 1 h at 30°C in the dark. The composition of the incubation buffer was 0.1 M $KNO_3$, 0.1 M $KH_2PO_4$, and 3% 1-propanol, and the pH was adjusted to about 7.5 with NaOH. Enzyme activity was stopped by placing sample vials in hot water (80°C). Leaves and roots were removed, oven-dried at 105°C, and then weighed to calculate the activity per unit dry weight. The concentration of $NO_2^-$-N produced in the incubation buffer was measured colorimetrically by diazotization[17]. The effect of plant pigment was compensated for by measurement of complete controls lacking N-naphtylethylene diamine dihydrochloride.

The remaining leaves and fine roots were dried at 40°C and then ground. About 100 mg of ground sample was extracted with 10 ml of deionized water for 1 h at 45°C. The extract was filtered, and the concentration of $NO_3^-$-N in the extract was analyzed by HPLC (SHIMADZU, HIC-6A, Kyoto, Japan) within 72 h to avoid the transformation of nitrate in the extract.

### Perlite Analysis

A 5-g subsample of perlite from each cultivation pot was extracted with 50 ml of 2 M KCl and filtered. The $NO_3^-$-N concentration in the extract was determined by diazotization after reduction of $NO_3^-$-N to $NO_2^-$-N with zinc powder[17]. The amount of $NO_3^-$-N retained in perlite was calculated as N per 100 ml core (µmol N 100 ml$^{-1}$) and compared with the data of the $NO_3^-$-N pool size in the forest soil where seeds of *L. triloba* were collected (Koyama, unpublished data). In the forest, 30 soil samples were collected from areas within a 30-cm radius from ten trunks of *L. triloba* in Mt. Ryuoh; this process was repeated five times during the 1998 growing season. A total of 150 soil samples were measured to determine $NO_3^-$-N pool sizes in the forest.

### Statistical Analysis

All statistical analyses were conducted using the statistical program SPSS 7.5.1[18]. Differences among NRAs or $NO_3^-$-N concentrations in plants supplied with different concentrations of $NO_3^-$-N were analyzed using a Kruskal–Wallis one-way analysis of variance. Multiple comparisons of mean values among treatments were performed by the sequential Bonferroni test[19] after the determination of pairwise P values by the Mann–Whitney test. In cases where multiple comparisons indicated that saturation had occurred in the relation between supplied $NO_3^-$-N and NRA or $NO_3^-$-N concentrations in the plants, Michaelis–Menten kinetics was applied for the relation of supplied $NO_3^-$-N and plant NRA or $NO_3^-$-N concentration as follows[20]:

$$v = S \times V_{max}/(S + Km)$$

where v is plant NRA or $NO_3^-$-N concentration, S is the concentration of supplied $NO_3^-$-N, Vmax is maximum value, and Km is the Michaelis constant. The two parameters, Vmax and Km, in the Michaelis–Menten kinetics were estimated by an Eadie–Hofstee plot (i.e., the relation of supplied $NO_3^-$-N to supplied $NO_3^-$-N/NRA)[21], and they were applied as initial values in the nonlinear regression analysis in SPSS. Spearman rank correlation coefficients were calculated to detect a relationship between NRA and $NO_3^-$-N concentration in each of plant leaves and roots. Spearman rank correlation coefficients were also calculated to detect a relationship between leaves and roots for each of NRA and $NO_3^-$-N concentration.

## RESULTS

### Plant NRA and $NO_3^-$-N Concentration

Root NRA changed with $NO_3^-$-N supply in the range from 0.071 to 1.79 mmol N $l^{-1}$ supplied $NO_3^-$-N (Fig. 1a); however, there was no significant difference between root NRA of individuals supplied with 1.79 and 3.57 mmol N $l^{-1}$ $NO_3^-$-N, indicating that root NRA had reached a plateau. The nonlinear regression analysis yielded values of Vmax = 0.46 (µmol N g dry $wt^{-1}$ $h^{-1}$) and Km = 1.33 (mmol N $l^{-1}$) for the relationship between root NRA and $NO_3^-$-N supply. In contrast, leaf NRA remained low even at the highest concentration of $NO_3^-$-N, and there was no significant difference among treatments. Root $NO_3^-$-N concentrations increased with $NO_3^-$-N supply (Fig 1c); however, leaf $NO_3^-$-N concentrations remained low with increased $NO_3^-$-N supply (Fig 1d), even though the $NO_3^-$-N concentrations in leaves were higher than in roots when the concentration of supplied $NO_3^-$-N was lower than 0.071 mmol N $l^{-1}$ ($p < 0.01$).

Comparisons of results between roots and leaves showed that there was no significant correlation between root NRA and leaf NRA or between root $NO_3^-$-N concentration and leaf $NO_3^-$-N concentration (Fig. 2). There was no significant correlation between leaf $NO_3^-$-N concentration and leaf NRA, although root NRA was significantly correlated with root $NO_3^-$-N concentration ($p < 0.01$) (Fig. 3).

### Perlite $NO_3^-$-N

The amount of $NO_3^-$-N retained in perlite increased from 0 up to 154.26 µmol N 100 ml $perlite^{-1}$ and was significantly correlated with the $NO_3^-$-N supply ($p < 0.001$) (Fig. 4a). Using the regression of supplied $NO_3^-$-N to retained $NO_3^-$-N in perlite, the perlite supplied with 2.06 mmol N $l^{-1}$ $NO_3^-$-N was equal to the maximum $NO_3^-$-N pool size in the forest soil (79.47 µmol N 100 ml $soil^{-1}$, Fig. 4b).

## DISCUSSION

### Effects of $NO_3^-$-N Supply on $NO_3^-$-N Use by *L. triloba*

When the concentration of supplied $NO_3^-$-N was lower than 1.79 mmol N $l^{-1}$, NRA in *L. triloba* roots increased with $NO_3^-$-N supply (Fig. 1a). Because there was no significant difference between root NRA supplied with 1.79 mmol N $l^{-1}$ and with 3.57 mmol N $l^{-1}$, it is likely that root NRA was saturated with 1.79 mmol N $l^{-1}$of $NO_3^-$-N. Two possible explanations can be considered for the control of root NRA by $NO_3^-$-N: (1) limited uptake of $NO_3^-$-N and (2) limited induction of NR by $NO_3^-$-N after it is taken up. The concentration of $NO_3^-$-N in plant organs is the difference between increase of $NO_3^-$-N by uptake and decrease of $NO_3^-$-N by reduction, as plants do not synthesize $NO_3^-$-N[11]; therefore, $NO_3^-$-N concentration in plant organs must be less than or equal to the $NO_3^-$-N absorbed by the plant. Nonetheless, root $NO_3^-$-N concentrations continuously increased with $NO_3^-$-N supply, showing no plateau (Fig. 1c). This indicates that the saturation of root NRA was not caused by the limited absorption of $NO_3^-$-N, although there was a significant correlation between root $NO_3^-$-N concentration and root NRA ($p < 0.01$) (Fig. 3a). This suggested that the $NO_3^-$-N uptake by *L. triloba* corresponds to the $NO_3^-$-N supply, even though the $NO_3^-$-N utilization of this species did not correspond to the absorbed $NO_3^-$-N when excess amounts of $NO_3^-$-N were supplied.

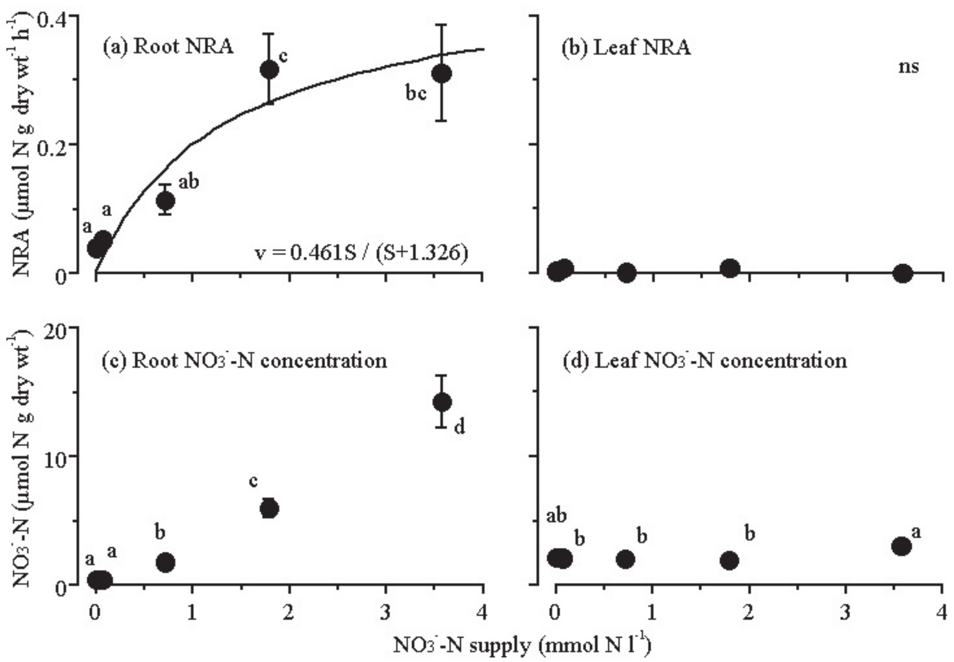

**FIGURE 1.** Effect of $NO_3^-$-N supply on (a) root NRA (µmol N g dry $wt^{-1}$ $h^{-1}$), (b) leaf NRA (µmol N g dry $wt^{-1}$ $h^{-1}$), (c) root $NO_3^-$-N concentration (µmol N g dry $wt^{-1}$), and (d) leaf $NO_3^-$-N concentration (µmol N g dry $wt^{-1}$). The curved line shown in (a) shows the Michaelis–Menten kinetics. The bars show S.E.

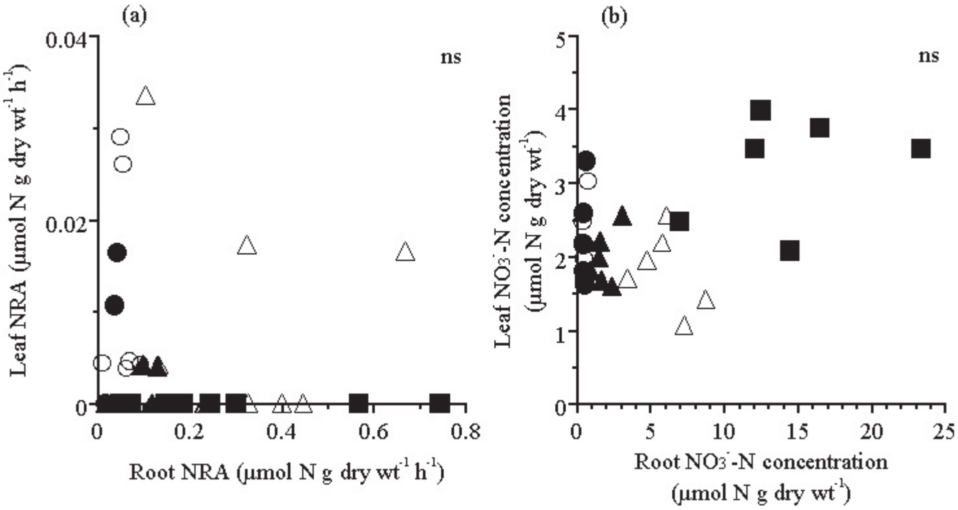

**FIGURE 2.** Relationship between (a) root NRA (μmol N g dry wt$^{-1}$ h$^{-1}$) and leaf NRA (μmol N g dry wt$^{-1}$ h$^{-1}$) and (b) root $NO_3^-$-N concentration (μmol N g dry wt$^{-1}$) and leaf $NO_3^-$-N concentration (μmol N g dry wt$^{-1}$). Concentrations of supplied $NO_3^-$-N were 0 (●), 0.071 (○), 0.71 (▲), 1.79 (△), and 3.57 (■) mmol N l$^{-1}$.

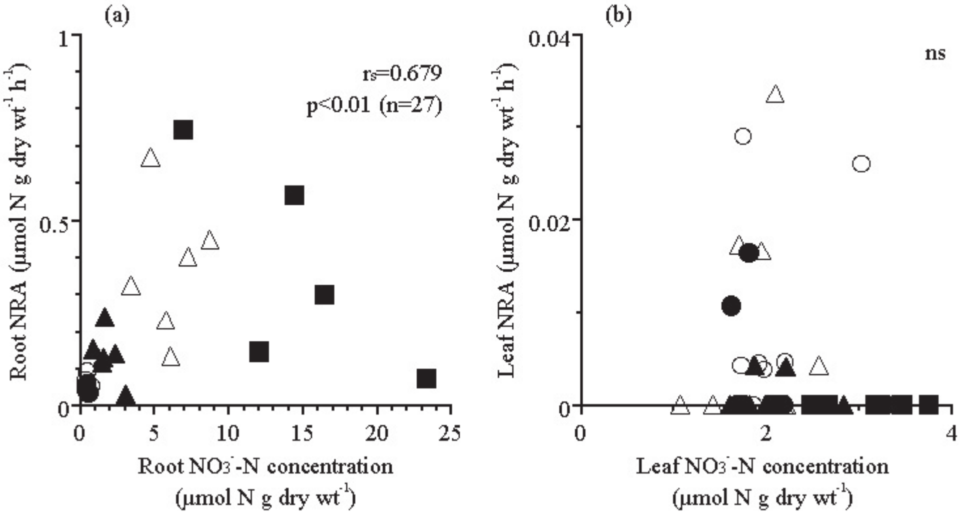

**FIGURE 3.** Relationship between (a) root $NO_3^-$-N concentration (μmol N g dry wt$^{-1}$) and root NRA (μmol N g dry wt$^{-1}$ h$^{-1}$) and (b) leaf $NO_3^-$-N concentration (μmol N g dry wt$^{-1}$) and leaf NRA (μmol N g dry wt$^{-1}$ h$^{-1}$). Concentrations of supplied $NO_3^-$-N were 0 (●), 0.071 (○), 0.71 (▲), 1.79 (△) and 3.57 (■) mmol N l$^{-1}$.

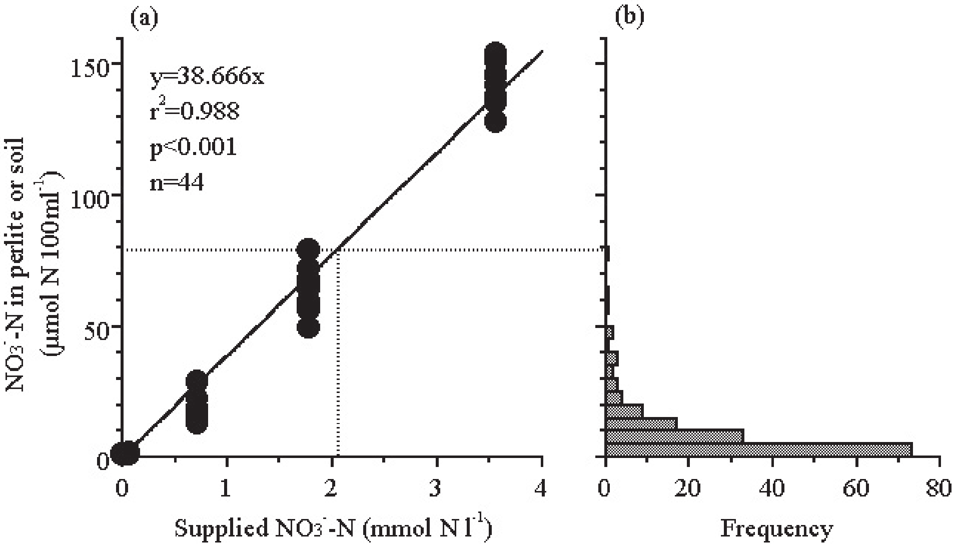

**FIGURE 4.** Comparison of $NO_3^-$-N content in perlite and forest soil. (a) Relationship between $NO_3^-$-N supply (mmol N l$^{-1}$) and amount of retained $NO_3^-$-N in perlite (μmol N 100ml$^{-1}$). (b) Frequency distribution for soil $NO_3^-$-N pool size (μmol N 100 ml$^{-1}$) in the forest where the seeds were collected (Koyama, unpublished data). The dotted lines connecting figures indicated (1) the maximum of $NO_3^-$-N pool size in forest soil and (2) the corresponding $NO_3^-$-N supply to achieve that maximum.

On the other hand, there was no significant difference in leaf NRA supplied with different concentrations of $NO_3^-$-N (Fig. 1b). Moreover, mean leaf NRA values were constantly lower than root NRA, irrespective of supplied $NO_3^-$-N concentration ($p < 0.001$). Two reasons can be offered for very low NRA in leaves compared with roots: (1) the lack of enzyme induction in leaves and (2) the lack of $NO_3^-$-N transportation from roots to leaves. The former reason, however, is not plausible because an investigation on *L. triloba* naturally grown in a conifer plantation showed that this species has NRA in its leaves, and the activity was approximately comparable to root NRA detected in this study (Koyama, unpublished data); therefore, it is obvious that *L. triloba* is able to induce NR in its leaves when $NO_3^-$-N is transported to the leaves. Besides leaf NRA, leaf $NO_3^-$-N concentrations also remained low even when a high concentration of $NO_3^-$-N was supplied, and root $NO_3^-$-N concentration showed no relationship with leaf $NO_3^-$-N concentration (Figs. 1d and 2b). The lack of a significant correlation between leaf NRA and leaf $NO_3^-$-N concentration can be ascribed to the narrower range of NRA and $NO_3^-$-N concentrations in leaves than in roots (Fig. 3b); therefore, leaves of *L. triloba* may play only minor part in $NO_3^-$-N use in the case of seedlings, and it may be because $NO_3^-$-N absorbed by roots was not transported to the leaves. The transportation of $NO_3^-$-N in plants and the allocation of NRA are influenced by factors such as specific property, light condition, external $NO_3^-$-N availability, plant age, and/or temperature[7,8,22,23,24,25,26]. Specific differences and light conditions cannot explain the absence of (or very low) leaf NRA in the present study. It is because the same species showed foliar NRA in field investigations as stated above; and the light availability must be higher in the cultivation experiment than under the field conditions in the conifer plantation, though it is commonly accepted that the better light conditions provide plants an advantage in leaf $NO_3^-$-N reduction[7,25]; however, further information is required to clarify the effects of other possible factors.

Moreover, when the concentrations of supplied $NO_3^-$-N were lower than 0.071 mmol N $l^{-1}$, $NO_3^-$-N, concentrations were significantly higher in leaves than in roots ($p < 0.01$), even though NRA was significantly lower in leaves than in roots across all levels of supplied $NO_3^-$-N ($p < 0.01$). This result suggests that *L. triloba* has a storage pool of $NO_3^-$-N in its leaves separate from the site of metabolism, and the $NO_3^-$-N transported into the storage pool cannot be assimilated. It could, however, play a part in ionic and osmotic balance in the cells[27].

## Estimation of $NO_3^-$-N Availability in Forest Soil to $NO_3^-$-N Use by *L. triloba*

Because the supplied solution (200 ml) overflowed the seedling receptacles, the $NO_3^-$-N available to *L. triloba* was equivalent not to the total amount of added $NO_3^-$-N but to the amount of $NO_3^-$-N retained in perlite. Among the treatments, however, the amount of $NO_3^-$-N retained in perlite was significantly correlated with the concentration of supplied $NO_3^-$-N ($p < 0.001$) (Fig. 4a); therefore, the range of $NO_3^-$-N pool size in forest soils (from 0 to 79.47 µmol N 100 ml soil $^{-1}$) was equivalent to the amount of $NO_3^-$-N retained in perlite supplied with $NO_3^-$-N of 0 to 2.06 mmol N $l^{-1}$ from the regression. The substitution of the maximum $NO_3^-$-N pool size in forest soil (namely, supply of 2.06 mmol N $l^{-1}$ $NO_3^-$-N) for the Michaelis–Menten kinetics gives 0.28 µmol N g dry wt$^{-1}$ h$^{-1}$ in NRA, which is equivalent to 60.8% of maximum value (0.46 µmol N g dry wt$^{-1}$ h$^{-1}$). Because there was no significant difference between the seedlings supplied with 1.79 mmol N $l^{-1}$ $NO_3^-$-N and with 3.57 mmol N $l^{-1}$ $NO_3^-$-N (Fig. 1a), root NRA might almost reach the plateau when 2.06 mmol N $l^{-1}$ $NO_3^-$-N (that is equivalent to the maximum $NO_3^-$-N pool size in forest soils) was supplied; however, as the frequency distribution for soil $NO_3^-$-N pool size was positively skewed (Fig. 4b), 90% of forest soils had a smaller $NO_3^-$-N pool size than perlite supplied with 0.71 mmol N $l^{-1}$ $NO_3^-$-N. When $NO_3^-$-N availability is in this range, roots of *L. triloba* are likely have a value of NRA lower than 0.16 µmol N g dry wt$^{-1}$ h$^{-1}$ (35.0% of maximum), assuming the preceding Michaelis–Menten kinetics apply. Moreover, root NRA increased with $NO_3^-$-N supply across this range, suggesting that $NO_3^-$-N availability is the limiting factor for $NO_3^-$-N assimilation by *L. triloba* grown in forest soils. These comparisons between $NO_3^-$-N retained in perlite and $NO_3^-$-N in forest soils suggest that available $NO_3^-$-N in forest soils is below the saturation concentration for *L. triloba*.

## ACKNOWLEDGMENTS

We would like to thank H. Takeda, N. Osawa, and T. Osono for their valuable advice. We thank Y. Asano and M. Katsuyama for their valuable advice in HPLC analysis, K. Ishimaru and other members of Laboratory of Forest Ecology, Kyoto University for their help in fieldwork and laboratory analysis, and H. J. Barclay for his linguistic help with the manuscript. We also appreciate the helpful suggestions from two anonymous reviewers and the help of the editors and the conference coordinators.

| | | |
|---|---|---|
| Received: | July | 10, 2001 |
| Revised: | November | 8, 2001 |
| Accepted: | November | 9, 2001 |
| Published: | November | 20, 2001 |
